# Opposing Roles of Testosterone and Cortisol in Prosocial Risk-Taking

**DOI:** 10.3390/bs16040568

**Published:** 2026-04-09

**Authors:** Shunhang Huang, Di Zhang, Jinying Zhuang

**Affiliations:** School of Psychology and Cognitive Science, East China Normal University, Shanghai 200241, China; 52193200017@stu.ecnu.edu.cn (S.H.); zhangdiskinner@163.com (D.Z.)

**Keywords:** prosocial risk-taking, testosterone, cortisol, dual-hormone hypothesis

## Abstract

Prosocial risk-taking is defined as engagement in altruistic behaviors that may have personal costs. Emerging research indicates that testosterone—a gonadal steroid hormone—is linked to behaviors aimed at promoting one’s social status. In line with these findings, we hypothesized that testosterone levels would be associated positively with prosocial risk-taking. Additionally, according to the dual-hormone hypothesis, this relationship may be moderated by cortisol. To examine these hypotheses, we administered an adapted version of the probabilistic gambling task, which included a prosocial condition. In the original task, participants were the beneficiaries of their choices; under the prosocial condition, the benefits were directed to charitable organizations. Our results revealed that, consistent with previous findings, the endogenous testosterone level was associated positively with risk-taking for personal gain. Notably, we also observed an association between the endogenous testosterone level and prosocial risk-taking. These relationships were not moderated by cortisol, meaning that the dual-hormone hypothesis was not supported. Instead, elevated cortisol independently suppressed prosocial risk-taking. Collectively, these results demonstrate that basal testosterone and cortisol levels play different roles in the modulation of prosocial risk-taking behavior.

## 1. Introduction

Prosocial risk-taking can be defined as risky behaviors undertaken with the primary intention of benefiting others rather than oneself, involving personal costs that may be social, physical, emotional, or otherwise (Do et al., 2017). Humans frequently engage in such acts of altruism that entail personal risks, ranging from extraordinary and courageous actions—such as voluntary enlistment in defense of one’s nation—to everyday scenarios, including advocating for marginalized acquaintances or mediating disputes among strangers. Despite the inherent hazards that may compromise their personal well-being, individuals often persist in such behaviors.

Previous studies have shown that prosocial risk-taking is positively associated with personality traits such as empathy and sensation-seeking (Armstrong-Carter et al., 2021). In addition, mating motivation has been found to increase men’s willingness to engage in prosocial risk-taking behaviors that allow them to display heroism or dominance (Griskevicius et al., 2007). Despite the prevalence and theoretical importance of prosocial risk-taking, the biological mechanisms underlying this behavior remain poorly understood.

One promising candidate is testosterone. A prominent steroid hormone synthesized by the gonads, it is widely acknowledged to play a pivotal role in the regulation of physiological and behavioral processes associated with survival and reproduction (Mooradian et al., 1987). It has also been associated with increased risk-taking behavior (Apicella et al., 2015). For example, in the Iowa Gambling Task, individuals with higher endogenous testosterone levels exhibited greater risk-taking tendencies (Singh, 2021). Similarly, in an asset trading game, men who received exogenous testosterone were more inclined to select riskier assets than those in the placebo group (Cueva et al., 2015).

Beyond general risk-taking, testosterone has also been shown to facilitate prosocial behaviors, particularly when such behaviors help attain or maintain a higher social status (Dreher et al., 2016; Eisenegger et al., 2010). For instance, in a modified Ultimatum Game where participants, acting as responders, could punish unfair proposers or reward fair ones at a personal monetary cost, exogenous testosterone administration significantly enhanced prosocial behavior by increasing the monetary rewards given to proposers who made fair or advantageous offers—actions interpreted as serving to bolster the player’s social status (Dreher et al., 2016).

According to the dual-hormone hypothesis (Mehta & Prasad, 2015), the impact of testosterone on social behavior is contingent upon cortisol, a hormone produced by the hypothalamic–pituitary–adrenal axis (Dickerson & Kemeny, 2004). Specifically, a positive correlation is observed between elevated testosterone levels and an enhanced inclination toward pursuing higher social status in the presence of low cortisol levels, rather than high levels (Prasad et al., 2019; Terburg et al., 2009). However, empirical support for this interaction remains inconsistent. For example, in a lottery task, the interaction between exogenous testosterone and basal cortisol level on risk-taking behavior was not significant (Nadler et al., 2024). Similarly, a meta-analysis of research related to the dual-hormone model indicated that dual-hormone effects are significant but weak and context-dependent (Dekkers et al., 2019).

In addition, cortisol may directly suppress prosocial risk-taking through two distinct pathways. First, it can increase risk aversion, thereby reducing willingness to engage in risky prosocial actions. For instance, administration of exogenous cortisol has been shown to increase risk aversion in decision-making tasks (Coates & Herbert, 2008). Second, cortisol may inhibit prosocial risk-taking by directly decreasing prosocial motivation. According to the conservation of resources theory (Hobfoll, 1989), individuals facing threats prioritize the protection of existing resources. Because acute stress depletes discretionary resources and prosocial acts require their expenditure, the theory predicts a decrease in prosocial behavior under stress. This prediction is supported by evidence linking elevated cortisol to reduced charitable donation (Schulreich et al., 2022).

The objectives of the present study were to examine the association between salivary baseline testosterone levels and prosocial risk-taking and to determine whether this relationship was mediated by cortisol. To investigate these relationships, we added a prosocial condition to the probabilistic gambling task (PGT; Smith et al., 2014). Our findings help to elucidate the neuroendocrine mechanisms underlying real-world altruistic behaviors, such as charitable giving, when individuals sacrifice personal resources for the benefit of others.

## 2. Materials and Methods

### 2.1. Participants

The sample size was determined a priori using G*Power 3.1 (Faul et al., 2007). We aimed to recruit a minimum of 68 participants to achieve approximately 80% statistical power to detect an effect size of *f* = 0.15. Seventy-four healthy male participants were recruited for this study and instructed to refrain from consuming alcohol, caffeine, and tobacco for 24 h before the testing session. This study was conducted in accordance with the Declaration of Helsinki and was approved by the local research ethics committee (no. HR2-0202-2021). All participants were free from mental disorders and had not used hormone drugs recently. They had normal or corrected-to-normal vision and provided informed consent prior to participation. Participants under the age of 18 obtained their parents’ informed consent. Each participant received a flat fee of 60 yuan and a bonus payment of 0–20 yuan, which was determined by the number of tokens they earned during the task. Data on three participants were lost due to a program crash, and two participants chose not to contribute to a public welfare project in a prosocial condition. Consequently, data from these five individuals were excluded, resulting in a sample of valid data from 69 participants [mean (M) age = 21.44 years, standard deviation (SD) = 2.69 years, age range = 17–29 years].

### 2.2. (Prosocial) Risk-Taking Behavior Assessment

To measure participants’ prosocial risk-taking behaviors, a PGT (Smith et al., 2014) was adapted to include a prosocial condition involving beneficiary assignment, in contrast to the original self-oriented condition wherein participants engage in risk-taking decisions with the objective of maximizing their individual gains. Under the prosocial condition, participants assume personal risks to benefit a designated charitable organization. Before starting, each participant selected a public welfare project from Alipay, a major Chinese smartphone payment platform, to be the beneficiary of their donations.

In each PGT trial, participants were presented with a wheel composed of pie-shaped red, green, and gray sections for 2000 ms. The wheel comprised six categories, each with a unique color ratio. The gray section consistently accounted for 10% of the area. The remaining area was divided between the red section (loss of 10 tokens) and the green section (gain of 10 tokens). The loss-to-gain probability ratio was defined as the proportion of the red area (loss) relative to the green area (gain), yielding six levels: 2.91 (36% red vs. 54% green), 2.00 (45% red vs. 45% green), 1.50 (50% red vs. 50% green), 1.25 (54% red vs. 36%), 1.00 (45% red vs. 45% green), and 0.67 (36% red vs. 54% green). A higher loss-to-gain ratio indicated a higher level of gambling risk, as the probability of losing tokens increased relative to the probability of gaining them. Participants were explicitly informed that the proportional size of each section directly corresponded to the actual probability of the outcome: landing on red resulted in a loss of 10 tokens, landing on green resulted in a gain of 10 tokens, and landing on gray resulted in no change (10 tokens = 1 yuan).

After the wheel presentation, the words “start” and “skip” appeared at the top of the wheel and the participant was required to make a selection within 1500 ms. A “j” key press indicated abstention from the gamble, and a screen with the words “no play” appeared. An “f” key press initiated the gambling; the wheel spun and eventually came to a halt with an indicator pointing toward one of the three sections. After each trial, a feedback screen displayed the outcome (+10 for a win, −10 for a loss, and 0 for a neutral outcome or pass).

In both the self-oriented (original) condition and the prosocial condition, if the outcome was a loss, participants lost 10 tokens of their own. If the outcome was a win, participants earned 10 tokens, which were kept for themselves in the self-oriented condition but donated to their chosen charity in the prosocial condition.

Each participant performed 12 practice trials before starting the task. The experiment consisted of 84 trials each under the self-oriented and prosocial conditions. These trials were presented in a completely randomized order, with 14 trials involving each of the six loss-to-gain probability ratios. The order of the two conditions was counterbalanced across participants using an ABBA design. The task took about 40 min to complete.

### 2.3. Saliva Samples and Hormone Assays

To mitigate the influence of the salivary testosterone secretion rhythm on the data, saliva samples were collected from all subjects daily between 2:00 p.m. and 8:00 p.m. using the passive drooling method for the measurement of the testosterone and cortisol levels (Campbell et al., 1982). In each case, 1.8 mL of saliva was collected into a test tube. The participants were instructed to abstain from eating, smoking, and drinking coffee, tea, or milk tea for 1 h and to abstain from consuming alcohol for 4 h prior to sample collection.

The saliva samples were stored at −20 °C and subsequently transported to the testing facility for analysis using salivary testosterone enzyme-linked immunosorbent assay kits (DRG International, Marburg, Germany). The assay has a limit of detection of 7.1 pg/mL, with inter- and intra-assay coefficients of variation below 6.06% and 13.62%, respectively.

### 2.4. Statistical Analyses

The assays were performed in two batches. As testosterone levels were significantly lower in assay 2 (M = 56.420, SD = 36.377) than in assay 1 [M = 165.115, SD = 53.936; *t*(67) = 9.923, *p* < 0.01]. As the experimental location and storage conditions were consistent, this marked difference may be attributable to seasonal variations in testosterone levels, as assay 1 was performed in November (late autumn/early winter) and assay 2 in September (early autumn/lingering summer), aligning with patterns observed in East Asian populations where testosterone tends to be relatively lower in early autumn (still influenced by residual summer heat and humidity) and higher in late autumn/early winter as temperatures cool (Lee & Lee, 2021; Sim et al., 2017). To mitigate this batch effect, raw testosterone data from each assay were analyzed separately (Stanton et al., 2011).

We standardized the testosterone concentrations by converting raw scores to *z* scores for each assay and participant. As in previous studies (Mehta & Josephs, 2010; Stanton et al., 2021), the cortisol distribution was skewed in our study; we thus log-transformed the cortisol data.

To examine the relationship between testosterone levels and risk-taking behavior, we conducted a generalized linear mixed model analysis (GLMM) using the lme4 package (Bates et al., 2014) in R 4.5.0. The model included the task condition (self-oriented vs. prosocial), loss-to-gain probability ratio, testosterone levels, cortisol levels, and their interaction terms as predictors, with risk-taking behavior serving as the outcome variable. *p* values < 0.05 were considered significant.

## 3. Results

### 3.1. Main Effects

**Figure 1:**
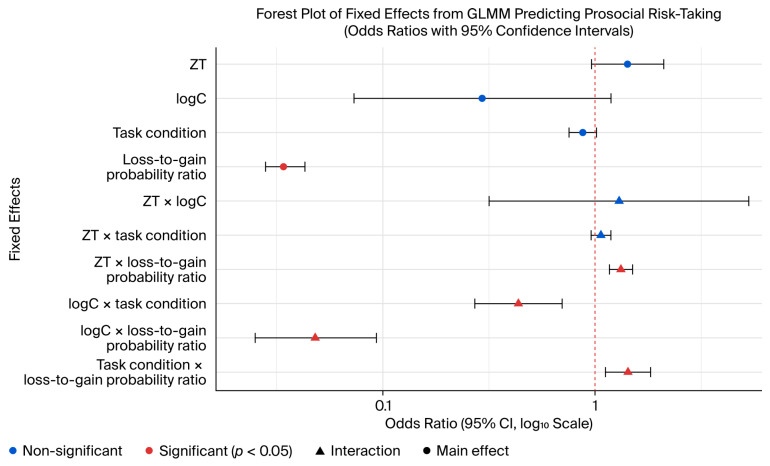
Forest plot displaying odds ratios (ORs) and 95% confidence intervals (CIs) for fixed effects and interactions from the GLMM predicting risk-taking choices. Predictors include standardized testosterone (ZT), task condition (self-oriented vs. prosocial), loss-to-gain probability ratio, and log-transformed cortisol (logC).

The results of the GLMM predicting risk-taking choices are summarized in Table 1 (random coefficient models across three specifications) and Figure 1 (forest plot of odds ratios and 95% confidence intervals for all fixed effects and interactions).

**Figure 2:**
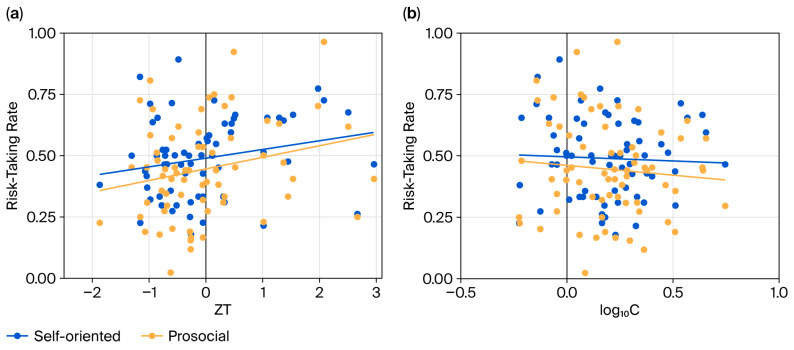
Hormonal effects on risk-taking behaviors. Associations of the testosterone–cortisol ratio (ZT, standardized testosterone) (**a**) and log-transformed cortisol (log_10_C) (**b**) with risk-taking rates under the self-oriented and prosocial behavioral conditions.

As shown in Table 1 (Model 1), the GLMM revealed a marginally significant positive main effect of testosterone (*b* = 0.353, standard error (SE) = 0.200, 95% CI [0.962, 2.106], *z* = 1.767, *p* = 0.077); higher testosterone levels predicted increased risk-taking behavior across both conditions (Figure 2a). A marginally significant negative main effect was observed for cortisol (*b* = −1.224, SE = 0.713, 95% CI [0.073, 1.189], *z* = −1.718, *p* = 0.086). A significant main effect of the task condition was also observed, with participants engaging in less risk-taking under the prosocial condition than under the self-oriented condition (*b* = −0.134, SE = 0.076, 95% CI [0.753, 1.016], *z* = −1.749, *p* = 0.080). Furthermore, higher gain-to-loss probability ratios were associated with markedly reduced risk-taking willingness (*b* = −3.371, SE = 0.110, 95% CI [0.028, 0.043], *z* = −30.577, *p* < 0.001).

### 3.2. Interaction Effects

**Figure 3:**
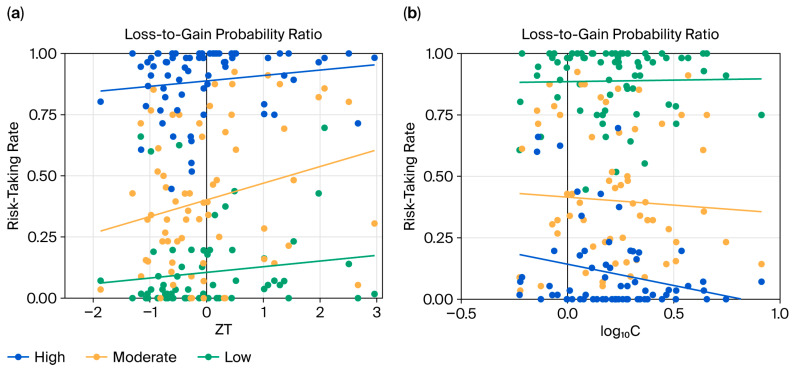
Hormonal effects on risk-taking behaviors. Associations of the testosterone–cortisol ratio (ZT, standardized testosterone) (**a**) and log–transformed cortisol (log_10_C) (**b**) with risk-taking rates under high (2.91 and 2), moderate (1.5 and 1.25), and low (1 and 0.67) loss-to-gain probability ratios.

Significant interaction between the testosterone level and the gain-to-loss probability ratio was observed (*b* = 0.345, SE = 0.062, 95% CI [0.271, 0.699], *z* = 5.600, *p* < 0.001; Figure 3a). Simple effects analysis confirmed that testosterone exerted a strong influence at high (*b* = 0.665, SE = 0.197, *z* = 3.380, *p* < 0.001) and moderate (*b* = 0.457, SE = 0.193, *z* = 2.37, *p* < 0.001) probability ratios, but not at low ratios (*p* = 0.202). The interaction between testosterone and the task condition was not significant (*p* = 0.349).

A significant interaction emerged between cortisol levels and task conditions (*b* = −0.833, SE = 0.242, 95% CI [0.271, 0.699], *z* = −3.437, *p* < 0.001; Figure 2b). Specifically, the inhibitory effect of cortisol on risk-taking was stronger under the prosocial condition (*b* = −2.057, SE = 0.715, *z* = −2.878, *p* = 0.004) than under the self-oriented condition (*b* = −1.224, SE = 0.712, *z* = −1.718, *p* = 0.086).

The interaction between the cortisol level and the loss-to-gain probability ratio was significant (*b* = −3.034, SE = 0.337, 95% CI [0.025, 0.093], *z* = −8.998, *p* < 0.001; Figure 3b). The dampening effect of cortisol was more pronounced under high (*b* = −5.335, SE = 0.880, *z* = −6.064, *p* < 0.001) than under moderate (*b* = −1.309, SE = 0.713, *z* = −1.834, *p* = 0.067) and low (*p* = 0.190) loss-to-gain ratios.

Significant interaction between the task condition and the loss-to-gain probability ratio was also observed (*b* = 0.282, SE = 0.064, 95% CI [1.119, 1.826], *z* = 4.409, *p* < 0.001). Simple effects analyses indicated that the effect of the condition was more pronounced at high (*b* = −0.735, SE = 0.221, *z* = 3.329, *p* < 0.001) probability ratios than at moderate (*b* = 0.361, SE = 0.200, *z* = 1.806, *p* = 0.071) and low (*p* = 0.460) loss-to-gain ratios.

Finally, the interaction between the testosterone and cortisol levels was not significant (*p* = 0.638).

Additionally, given the substantial differences between Assay 1 and Assay 2, we recalculated the main analyses using only data from Assay 1 and obtained essentially the same results for testosterone (see electronic Supplementary Materials, Table S1 for detailed regression outputs).

## 4. Discussion

In the current investigation, we explored the potential influence of testosterone levels on prosocial risk-taking behavior. To achieve this goal, we adapted a PGT (Smith et al., 2014), introducing a prosocial condition wherein participants assumed personal risks to benefit a charitable organization. The findings support the hypothesized link between personal and prosocial risk-taking (Do et al., 2017), confirming that individuals who take risks for self-gain are also more inclined to do so for others. Importantly, endogenous testosterone levels were associated positively with risk-taking propensity in both the personal and prosocial contexts, a relationship that strengthened with increasing loss-to-gain probability ratios. In contrast, cortisol levels were associated inversely with risk-taking across conditions, an effect that became more pronounced as the loss-to-gain probability ratio increased. Furthermore, the inhibitory effect of cortisol on risk-taking was more substantial under the prosocial condition than in the personal risk setting.

The observed positive association between endogenous testosterone levels and economic risk-taking behavior is consistent with the previous finding that individuals with higher salivary testosterone levels exhibited a stronger propensity for risk-taking in an investment game (Apicella et al., 2008). However, limited or inconsistent effects of testosterone on risk-taking have also been reported, with a meta-analysis revealing only a weakly significant correlation (Kurath & Mata, 2018). For instance, in a task where participants chose between a sure option with a lower reward and a risky lottery (50% chance of a higher reward or nothing), exogenous testosterone did not increase risk-taking propensity compared to a placebo (Nadler et al., 2024). Such discrepancies may arise from variation in the risk probability parameters used across experiments. Importantly, our data suggest that testosterone’s influence is particularly heightened in high loss probability contexts. Further research is needed to elucidate this relationship under diverse probabilistic conditions.

Notably, we found no evidence that testosterone differentially influenced self-oriented versus prosocial risk-taking. This pattern suggests that testosterone may enhance prosocial risk-taking indirectly, primarily by increasing individuals’ general propensity for risk-taking, rather than by selectively boosting prosocial motivation per se. According to the social status hypothesis, testosterone facilitates prosocial behaviors, particularly when they serve to signal positive social information to an audience (Dreher et al., 2016; Eisenegger et al., 2010). For example, a recent fMRI study by Li et al. (2020) demonstrated that heightened testosterone levels correlated significantly with increased striatal activation when subjects were being observed by others. The striatum has been implicated in cost/benefit analysis related to social reputation (Izuma, 2012). However, the present study did not manipulate social signals associated with prosocial behavior. Future research should explicitly examine the role of testosterone in prosocial risk-taking by explicitly manipulating reputational cues, such as the presence of observers.

In the present study, we did not find evidence that cortisol moderated the relationship between testosterone and either self-oriented or prosocial risk-taking. This null result may reflect the highly context-dependent nature of the dual-hormone hypothesis (Dekkers et al., 2019). For example, Knight et al. (2022) showed that the interactive effect of testosterone and cortisol on competitive behavior depended on opponent cues. Among men with low baseline cortisol, exogenous testosterone increased the preference for competing against male opponents or past winners while decreasing it against female opponents. In contrast, among men with high baseline cortisol, exogenous testosterone showed the reverse pattern: it increased the preference for competing against female opponents or past losers while decreasing it against male opponents. These considerations suggest that the absence of interaction in the current findings should be interpreted with caution.

With respect to the main effect of cortisol, its inhibitory influence on risk-taking was stronger in the prosocial condition than in the self-oriented condition. According to the conservation of resources theory (Hobfoll, 1989), cortisol tends to reduce engagement in behaviors that could lead to resource depletion, such as risk-taking and prosocial actions. For example, chronic cortisol elevation increases risk aversion, leading individuals to favor low-risk options (Kandasamy et al., 2014). Additionally, Schulreich et al. (2022) showed that acute stress-induced rises in cortisol significantly reduced charitable donations among individuals with high mentalizing abilities, an effect mediated by reduced neural value coding in the right dorsolateral prefrontal cortex. However, alternative explanations should be considered. The stronger inhibitory effect of cortisol in the prosocial condition might also reflect a generalized increase in risk avoidance or uncertainty aversion, rather than a specific depletion of prosocial motivation. Cortisol has been linked to heightened sensitivity to potential losses (Kandasamy et al., 2014). Without independent measures of stress, anxiety, or cognitive load, we cannot directly differentiate between motivational (e.g., resource conservation) and affective (e.g., anxiety-driven avoidance) pathways. Thus, while our findings are consistent with the conservation of resources framework, other interpretations remain plausible. Future studies should incorporate direct assessments of perceived resource depletion, state anxiety, and risk preferences to disentangle these mechanisms.

We acknowledge some limitations of our study. First, the relatively small sample size may have limited the statistical power to detect subtle effects, including potential testosterone × cortisol interactions. Second, the current study was performed with exclusively male participants. Given the sex-specific effects of hormonal processes on prosocial behavior (Eagly, 2009) and risk-taking (Kurath & Mata, 2018), further research is needed to determine whether these findings can be extrapolated to female populations. Third, relying on a single saliva sample limits the reliability of basal hormone estimation (Dabbs, 1990). Future studies could enhance reliability by using multiple measurements, ideally combining samples taken several weeks apart. Fourth, although the present study provides primary evidence on the correlation between testosterone levels and prosocial risk-taking, this evidence is only correlative. Further investigations involving exogenous testosterone administration are imperative to elucidate the causal role of testosterone in prosocial risk-taking. Finally, cortisol was assessed only at baseline, limiting insights into dynamic stress responses. According to prior research, testosterone–cortisol interactions may be more pronounced under conditions involving acute stress reactivity (Knight et al., 2020). Thus, the present study may not be optimally designed to test the dual-hormone hypothesis. Future investigations could strengthen causal inference by experimentally manipulating cortisol levels, for instance, through acute stress paradigms such as the Trier Social Stress Test (Kirschbaum et al., 1993).

## 5. Conclusions

The findings of this study indicate that elevated endogenous testosterone levels are associated positively with prosocial risk-taking behavior, which may stem primarily from testosterone’s facilitative effect on self-oriented risk-taking. In contrast, higher endogenous cortisol levels correlated negatively with prosocial risk-related decision-making, potentially mediated by cortisol’s modulation of risk perception and prosocial tendencies. These results provide initial insights into the hormonal regulatory mechanisms of prosocial risk-related decision-making, suggesting that testosterone and cortisol influence socially oriented decisions through distinct pathways.

## Figures and Tables

**Table 1:** Regression results using random coefficient modeling.

Variable	Risk-Taking Rate		
	Model 1	Model 2 (Without LogC)	Model 3 (Without ZT)
Intercept	−0.246	−0.451 **	−0.264
ZT	0.353	0.349 *	—
LogC	−1.224	—	−0.982
Loss-to-Gain Probability Ratio	−3.371 **	−3.911 ***	−3.401 ***
Task Condition ^a^	−0.134	−0.282 ***	−0.137
ZT × Loss-to-Gain Probability Ratio	0.282 **	0.211 **	—
ZT × Task Condition	0.063	0.039	—
ZT × LogC	0.260	—	—
LogC × Task Condition	−0.833 ***	—	−0.777 **
LogC × Loss-to-Gain Probability Ratio	−3.034 ***	—	−2.777 ***
Task Condition × Loss-to-Gain Probability Ratio	0.357 **	0.397 **	0.377 **
AIC	8517.73	8603	8533.77
BIC	8605.88	8661.77	8592.53
Log Likelihood	−4246.86	−4293.5	−4258.89

Note: N (observation) = 11,447. ZT: standardized testosterone; logC: log-transformed cortisol; AIC: Akaike information criterion; BIC: Bayesian information criterion. ^a^ 0 = self-oriented condition, 1 = prosocial condition. * *p* < 0.05, ** *p* < 0.01, *** *p* < 0.001.

## Data Availability

Data available in a publicly accessible repository.

## Supplementary Materials

The following supporting information can be downloaded at: https://www.mdpi.com/article/10.3390/bs16040568/s1, Table S1: Fixed effects from the GLMM predicting prosocial risk-taking using only Assay 1 data.
