# Supplementary material for: Opposing Roles of Testosterone and Cortisol in Prosocial Risk-Taking
Source: Behav Sci (Basel). 2026 Apr 9;16(4):568. doi: 10.3390/bs16040568 (PMC13113937; doi:10.3390/bs16040568)
Supplement: Supplementary file 1 [file behavsci-16-00568-s001.zip › behavsci-4144019-supplementary/supplementary material.pdf]

Table S1. Fixed effects from the GLMM predicting prosocial risk-taking using only Assay 1 data.

**Variable**	
Intercept	0.067
ZT	0.607 *
Task Condition	0.248 **
Loss-to-Gain Probability Ratio	3.135 ***
logC	1.148
ZT × Task Condition	0.007
ZT × Loss-to-Gain Probability Ratio	0.541 ***
ZT × logC	0.457
Task Condition × Loss-to-Gain Probability Ratio	0.182
Task Condition × logC	0.247
Loss-to-Gain Probability Ratio × logC	3.542 ***
AIC	5034.72
BIC	5116.6
Log Likelihood	-2505.36
